# LATENT CLASSES OF MULTIMORBIDITY AND ASSOCIATED CARE-RECEIVING CHARACTERISTICS FOR US OLDER ADULTS

**DOI:** 10.1093/geroni/igad104.2654

**Published:** 2023-12-21

**Authors:** Mona Liu, Corey Nagel, Siting Chen, Jeffrey Kaye, Heather Allore, Ana Quiñones

**Affiliations:** Oregon Health & Science University, Portland, Oregon, United States; University of Arkansas for Medical Sciences, Little Rock, Arkansas, United States; Oregon Health & Science University, Portland, Oregon, United States; Oregon Health & Science University, Portland, Oregon, United States; Yale University, New Haven, Connecticut, United States; Oregon Health & Science University, Portland, Oregon, United States

## Abstract

Older adults with different multimorbidity combinations require different intensities and types of care. This study aimed to identify subgroups of older adults based on their empirically-determined multimorbidity patterns, and used these to compare their care-receiving characteristics. Data from the National Health and Aging Trends Study (NHATS) in 2011 were utilized and ten chronic conditions were included to estimate latent classes among 7,532 individuals. A four-class solution was selected based on fit indices resulting in a relatively healthy (30%), cardiometabolic (25%), musculoskeletal (24%), and multisystem (21%) classes. The median number of caregivers was 2 for the multisystem class and 1 for the remaining three classes. Average total hours of care per month were 109.07 (SD=209.09), ranging from 94.43 (SD=168.71) for the relatively healthy class to 171.87 (SD=225.72) for the multisystem class. In assessing ADL and IADL domains of care received, the musculoskeletal class had the smallest proportion of people requiring care in all domains except for getting outside, dressing, and money management, even compared with the relatively healthy class. Substantially more people in the multisystem class required care in all domains except for money management. Sensitivity analysis using data from the refreshment cohort (NHATS 2015) corroborated the results. Results highlighted different care needs among persons with distinct combinations of multimorbidity, especially the vulnerability among people with multisystem multimorbidity and their caregivers. In addition, the findings called for further investigation into older adults characterized by musculoskeletal conditions to discern whether they had fewer care needs or more unmet needs.

